# Research progress of risk factors and early diagnostic biomarkers of gout-induced renal injury

**DOI:** 10.3389/fimmu.2022.908517

**Published:** 2022-09-20

**Authors:** Sheng Wang, Liyun Zhang, Dongsheng Hao, Lei Wang, Jiaxi Liu, Qing Niu, Liangyu Mi, Xinyue Peng, Jinfang Gao

**Affiliations:** ^1^ Shanxi Bethune Hospital, Shanxi Medical University, Taiyuan, China; ^2^ Department of Rheumatology, Shanxi Bethune Hospital, Shanxi Medical University, Taiyuan, China; ^3^ School of Basic Medicine, Shanxi Medical University, Taiyuan, China; ^4^ Third Hospital of Shanxi Medical University, Shanxi Bethune Hospital, Shanxi Academy of Medical Sciences, Tongji Shanxi Hospital, Taiyuan, China

**Keywords:** gout, gouty nephropathy, risk factors, early diagnosis, biomarkers

## Abstract

Gout renal injury has an insidious onset, no obvious symptoms, and laboratory abnormalities in the early stages of the disease. The injury is not easily detected, and in many cases, the patients have entered the renal failure stage at the time of diagnosis. Therefore, the detection of gout renal injury–related risk factors and early diagnostic biomarkers of gout renal injury is essential for the prevention and early diagnosis of the disease. This article reviews the research progress in risk factors and early diagnostic biomarkers of gout renal injury.

## 1 Introduction

Gout is a heterogeneous group of metabolic diseases caused by purine metabolic disorders, with hyperuricemia and resulting recurrent acute arthritis, chronic arthritis, joint deformity, presence of tophi, uric acid urolithiasis, and interstitial nephritis as clinical special diagnoses, and in severe cases, there is acute renal failure. Gout is divided into two categories: primary and secondary. Primary gout is more common and is mostly caused by abnormal purine metabolism. Secondary gout is associated with some systemic diseases or drugs (1). The prevalence of gout has steadily increased in the 20th century (2). The burden of gout increased globally between 1990 and 2017, with a standard prevalence of 7.9% (3). The prevalence of gout varies widely among ethnic groups and regions, with Asian and Hispanic populations having a 2.0% lower incidence of gout than African-American and Caucasian individuals (with a prevalence of 4.8%) in the United States (4). The prevalence is highest in Oceanic countries worldwide, particularly in specific ethnic groups, such as the native Taiwanese and Māori, where it is reported to be more than 10%, while it is lowest in developing countries. Gout is also common in Europe, with studies in France, Germany, Greece, Italy, the Netherlands, Spain, and the United Kingdom reporting a gout prevalence of 1%–4% between 2003 and 2014. For other European countries, studies of Scandinavian populations have found a gout prevalence varying from 0.02% to 1.8%, despite considerable heterogeneity in the duration of data collection, data source, and age of included subjects (5).

Gout is often accompanied by dyslipidemia, metabolic disorders, cardiovascular disease, and renal disease (6). Patients with hyperuricemia and gout are most likely to have affected kidneys, resulting in the formation of uric acid renal calculi and decreased renal function. Hyperuricemia and repeated gout attacks can damage the function of the kidneys, and impairment of renal function can further lead to hyperuricemia and gout attacks, which are mutually causal and closely related (7). Nearly all patients with gout can be found to have varying degrees of renal damage at autopsy, including glomerulosclerosis, interstitial nephritis, pyelonephritis, kidney stones, or renal insufficiency. Between 18% and 30% of patients with gout die of end-stage renal disease. At present, the treatment of uric acid nephropathy mainly involves lifestyle interventions, such as exercise, weight loss, eating less purine-rich meat, avoiding high-fructose intake, and urate-lowering drug therapy (8). If early diagnosis and appropriate treatment can be provided, the degree of renal disease can be greatly reduced or stopped. Therefore, it is particularly important to study the risk factors of gout and renal injury and their early diagnostic biomarkers. However, there are few reviews on this aspect. Here, we review recent studies on gout renal injury to provide some basis for preventing the occurrence and promoting the diagnosis of renal injury in patients with hyperuricemia and gout.

## 2 Gout kidney injury

### 2.1 Mechanism of gout kidney injury

Abnormal uric acid metabolism in the body due to increased production or decreased excretion will result in hyperuricemia; uric acid kidney stones will form when uric acid reaches a certain concentration. Soluble urate and urate crystals can damage the kidneys of patients with gout during disease progression.

#### 2.1.1 Normal metabolism of uric acid in the body

Uric acid plays a dominant role in the disease progression of gout, and it is mainly synthesized in the liver, intestines, and vascular endothelium as an end product of exogenous purines from food (100–200 mg/day), as well as endogenous purines from damaged dead cells (500–600 mg/day) (9). In other mammals, uric acid is further metabolized by uricase to allantoin. Because the human body lacks uricase (10), uric acid levels are far higher in humans than in other mammals. At physiological concentrations, urate is a powerful antioxidant that scavenges superoxide, hydroxyl radicals, and singlet oxygen. It also plays a neuroprotective role, maintains blood pressure, and stimulates the innate immune system (11). Recently, some studies have suggested that uric acid may provide a neuroprotective advantage in Parkinson’s disease (12) and schizophrenia (13) and may, therefore, serve as a biomarker for depression; SUA levels are lower in patients with depression than in normal controls (14).

The production and excretion of uric acid in healthy individuals are always maintained in a balanced state, and the kidney is the main regulator to maintain homeostasis. Studies have demonstrated that one-third of these uric acid excretions occur in the gastrointestinal tract and two-thirds in the kidneys. The classic model of uric acid excretion in the kidney consists of four steps: glomerular filtration (100%), pre-secretory reabsorption (98% to 100%), tubular secretion (50%), and post-secretory reabsorption (40%). Finally, approximately 8% to 12% of uric acid filtered by the glomeruli is excreted. Although this model is controversial, it is widely adopted. Major transporters associated with renal tubular reabsorption of uric acid are urate anion transporter 1 (URAT1), organic anion transporter 4 (OAT4), and glucose transporter 9 (GLUT9); urate excretion transporters include OAT1, OAT3, urate transporter (UAT), multidrug resistance protein 4 (MRP4/ABCC4), ABCG-2, and sodium-dependent phosphate transporters. The number of transporters affects serum uric acid levels to some extent (15). In addition, URAT1 has been shown to be regulated by α-protein kinase 1 (16), insulin (17), and the glucocorticoid receptor (18), which also partly explains the increased uric acid levels in some patients with diabetes and humans under stress conditions in clinical practice (19). In the gut, uric acid is converted to ammonia and carbon dioxide. Bacteria use intestinal ammonia for metabolism. It is now known that the ABCG2 locus is also involved in uric acid excretion into the gut (20).

#### 2.1.2 Mechanism of hyperuricemia

Hyperuricemia is defined based on the solubility of uric acid, that is, serum uric acid concentrations exceeding 7.0 mg/dL (416 μmol/L) (regardless of sex), as a result of excessive uric acid synthesis or insufficient intestinal and renal excretion (21), with abnormal renal metabolic uric acid function being the cause of hyperuricemia in 90% of individuals.

Increased uric acid synthesis can be seen in accelerated purine degradation, high-cell turnover (hemolysis, rhabdomyolysis, and tumor lysis), and fasting (22). Lesch–Nyhan syndrome is additionally caused by excess uric acid production due to hypoxanthine-guanine phosphoribosyltransferase deficiency (23). Hyperuricemia leads to gout and kidney stones, and it is also considered an indicator of diseases such as metabolic syndrome, diabetes, cardiovascular disease, and chronic kidney disease (24).

Hyperuricemia due to inadequate excretion is further divided into inadequate renal excretion and inadequate intestinal excretion. Insufficient renal uric acid excretion is caused by a combination of decreased glomerular filtration, decreased tubular secretion, and enhanced tubular reabsorption. As mentioned earlier, uric acid homeostasis is mainly affected by renal proximal tubular cells, which express several abnormal UATs causing hyperuricemia, and acute or chronic decreases in glomerular filtration can also lead to hyperuricemia. Proximal tubular reabsorption of uric acid is mainly controlled by URAT1, and this transport can be stimulated by reduced amounts of organic acids, drugs, and extracellular fluid, resulting in hyperuricemia (24). For example, URAT1 inhibitors, benzbromarone and probenecid, treat hyperuricemia by inhibiting renal reabsorption of urate (25). The urate excretion transporter, OT4, is located in the apical membrane of epithelial cells where it functions in uric acid reabsorption in the lumen through an intracellular dicarboxylate gradient (26). GLUT9 is a high-capacity UAT that accelerates uric acid reabsorption by transporting glucose (27). The urate excretion organic anion transporters, OAT1 and OAT3, expressed as OAT4, are also basolateral in the cell and have a large effect on urate excretion as urate/dicarboxylate exchangers (28, 29). Similarly, the gut is the main site of extrarenal urate excretion. In the mouse intestine, ABCG2 expression is predominantly located in the villous brush border of epithelial cells in the ileum and jejunum (30). Increased serum uric acid in patients with ABCG2 dysfunction may be explained by decreased intestinal urate excretion (31). ABCG2rs 2231142 has also been shown to be a risk allele for gout (32).

#### 2.1.3 Mechanism of uric acid nephrolithiasis

The incidence of uric acid nephrolithiasis is increasing worldwide and may be associated with an increasing prevalence of metabolic syndrome and diabetes. Over the past 30 years, uric acid nephrolithiasis has risen from 7% to 14% of total kidney stone formation (33). Low pH urine, hyperuricemia, and low urine output are the main factors in the formation of uric acid renal calculi. Together, these three risk factors increase the supersaturation of urine relative to free uric acid, leading to uric acid precipitation and stone formation.

Among these three risk factors, low urine pH is a major determinant of uric acid nephrolithiasis formation. Acidic urine protonates uric acid and forms uric acid nephrolithiasis. The significance of low urinary pH in the cause of uric acid stones has recently been further demonstrated in a Drosophila model of uric acid stone formation; knocking out the ortholog of the human Na/H exchange regulator in Drosophila results in too high acidity in the Malpighian tubules, which leads to the formation of uric acid crystals in the tubules (34). Healthy adults produce approximately 50 to 80 mEq of hydrogen ions daily, and if such large amounts of hydrogen ions appear in the urine without buffering, the urine pH will be very low, while, in fact, the urine pH is rarely lower than 4.5. This is because hydrogen ions are buffered by phosphate and ammonium ions before being excreted from the body. Approximately 70% of the hydrogen ions combine with ammonia to form ammonium ions. Ammonia is produced in the proximal tubules; acidosis and hypokalemia stimulate ammonia production, which combines with hydrogen ions to form ammonium in tubular epithelial cells or lumens. Excessive hydrogen ion production should be considered if ammonium excretion increases, such as in ketoacidosis and hyper-catabolism, and because of certain drugs such as salicylic acid and alcoholism. Another cause of impaired buffering in the kidney may be increased acid load, which is likely to originate in the gastrointestinal tract and liver. Decreased hepatic metabolism and increased production exacerbate renal acid load as a result of increased luminal secretion and absorption (35). One study showed a negative correlation between liver fat content and 24-h urine pH (36), which also demonstrates this view from the side.

Hyperuricosuria and low urine output increase the saturation of uric acid, and low urine output also promotes urine stasis and increases the risk of stone formation. In addition, uric acid calculi may also result from the metabolism of a variety of drugs that regulate uric acid excretion (e.g., benzbromarone and losartan) (37).

#### 2.1.4 Mechanism of renal injury induced by uric acid/uric acid crystals

**Figure 1:**
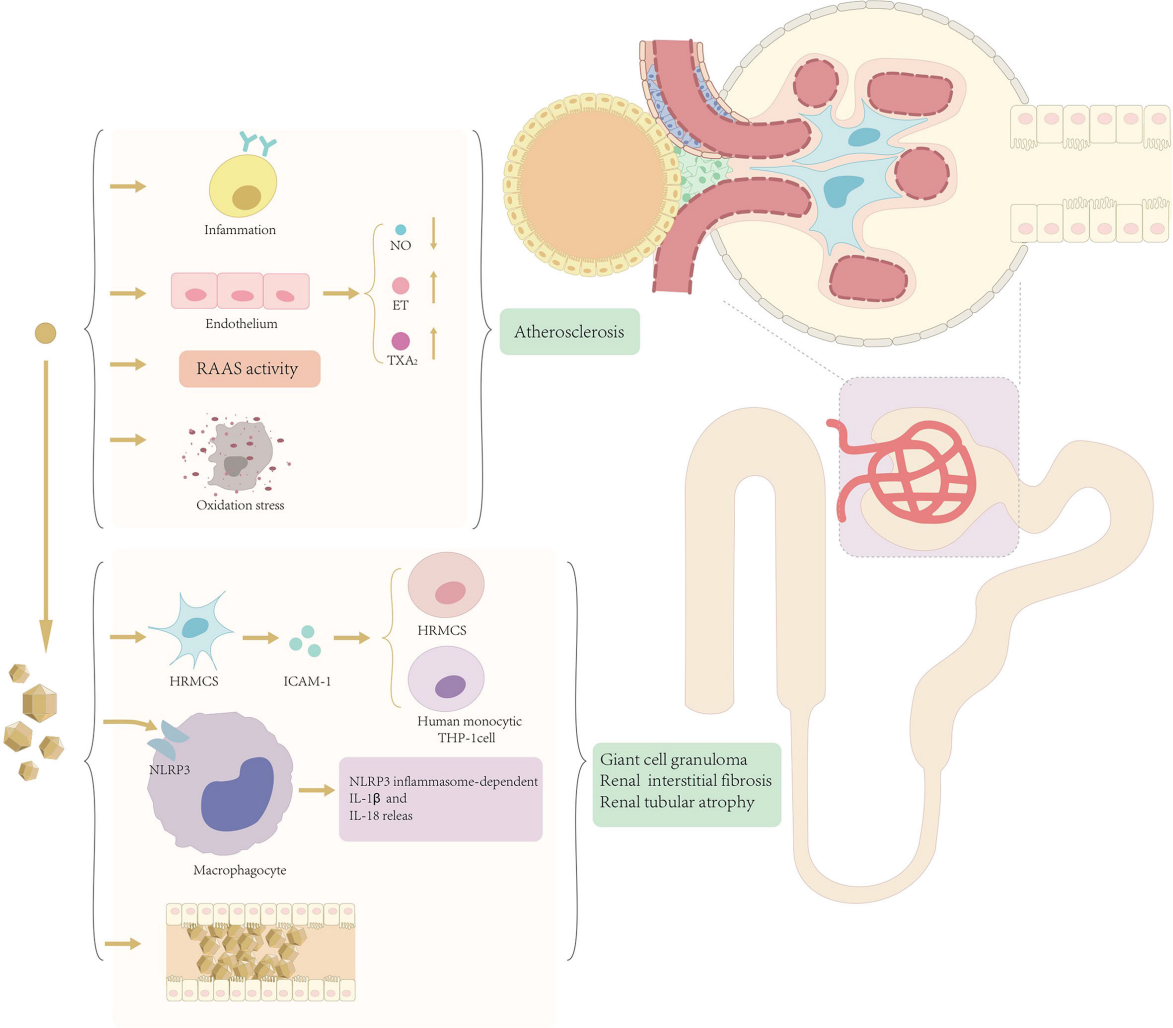
Mechanism of renal injury mediated by soluble uric acid and uric acid crystals.

At present, there are two different views on the mechanism of renal injury in patients with hyperuricemia and gout, i.e., the crystal mechanism of renal injury caused by urate crystals and the amorphous mechanism of renal injury caused by serum hyperuricemia (**Figure 1**).

##### 2.1.4.1 Crystal mechanism

Crystallographic mechanisms suggest that asymptomatic hyperuricemia does not lead to further development of renal injury unless uric acid forms urate crystals in the kidney (38). Uric acid crystals not only can directly cause renal obstruction but also can act as an intrinsic danger signal and activate a series of inflammatory responses in non-infected states.

Solubility studies have shown that serum supersaturates monosodium urate crystals when uric acid concentrations exceed 6.5 mg/dL (39), particularly in acidic urine (40). Urate crystals promote the development of gout kidney injury not only through extra neutrophil trapping nets (41) but also through the activation of the NALP3/IL-1β inflammasome pathway (42), and recent studies have also shown that urate crystals induce intercellular adhesion molecule-1 and intercellular adhesion in human mesangial cells (43). These together lead to the deposition of giant cell granulomas in the outer medullary crystals of the kidneys, resulting in renal interstitial fibrosis and tubular atrophy, and urate can also cause obstructive kidney disease by participating in uric acid stone formation, which in turn leads to a series of manifestations of decreased renal function.

##### 2.1.4.2 Amorphous mechanism

The amorphous mechanism suggests that deposition of urate crystals is usually focal and does not explain well the decline in the overall renal function. It is suggested that hyperuricemia can cause and accelerate the development of chronic kidney disease through hemodynamic and soluble uric acid effects on glomerular vascular and tubular cells. The specific mechanism is that hyperuricemia can lead to hypertension and renal impairment by stimulating the renin–angiotensin–aldosterone system (RAAS) and inhibiting nitric oxide synthesis and can also be involved in the development of renal disease by inducing inflammatory responses and oxidative stress (44). Hyperuricemia causes renal afferent renal arteriolopathy and tubulointerstitial fibrosis through the activation of the RAAS (45). It has been shown that blocking asymptomatic hyperuricemia by RAAS blockade adversely impacts the progression of chronic kidney disease and hypertension (46). Another study showed that decreased uric acid levels were accompanied by decreased blood pressure, which also suggests a direct or indirect relationship between hyperuricemia and hypertension (47). In addition to the RAAS, uric acid activates other vasoconstrictors, such as endothelin and thromboxane, and inhibits the vasodilator pathway, such as in nitric oxide (NO) release (48). Inducing systemic hypertension to the glomeruli promotes chronic kidney disease progression (49). This conclusion, although controversial, is supported by *in vitro* experimental studies showing that uric acid decreases NO production (50), and, in addition, experimentally induced urinary nitrite decreases in a rat model of hyperuricemia and leads to systemic and glomerular hypertension (51).

### 2.2 Clinical manifestations

Corresponding to the mechanism of renal injury caused by gout, renal injury caused by gout and hyperuricemia mainly involves the renal calculi and abnormal renal function. In addition, there is no definitive temporal sequence between gout renal injury and gouty arthritis in clinical practice, and some patients have history of renal calculi and decreased renal function before the onset of gouty arthritis, while some have renal impairment after gouty arthritis.

#### 2.2.1 Renal calculi

Patients with gout are at high risk of kidney stones. Microscopically, uric acid crystals are deposited in the renal papilla and collecting duct. Clinical manifestations are generally pain, manifesting as colic or dull pain, and the location of pain is mostly in the renal area or upper abdomen. In most cases, it is paroxysmal. Regarding hematuria, in most cases, it is microscopic and can be induced or aggravated after physical activity. It may accompany infection and obstruction. Regarding urinary excretion of sand stones, small stones may be excreted, some gritty; especially during pain and hematuria attacks, stones may be blocked or tingling may be felt when they pass through the urethra. Moreover, some patients with uric acid nephrolithiasis may have secondary urinary tract infections, often presenting with symptoms such as exotherm, chills, and bladder irritation. Obstruction: may cause hydrops above obstruction. Generally, stone obstruction is incomplete. The patient may experience urethral pain, dysuria, and urinary flow interruption during obstruction.

Urinary ultrasonography and abdominal computed tomography (CT) are helpful in the early diagnosis of renal calculi. On B-mode ultrasonography, urate deposits are hyperechoic, which allows us to recognize that this modality is also useful for detecting kidney stones and examining the renal medulla in patients with gout (52, 53). CT imaging is performed by measuring attenuation in Hounsfield units (HU) for stone composition analysis, and due to the different HU of each stone, there is considerable overlap between different stone types, limiting the usability of CT for this examination. Being able to distinguish the stone composition at diagnosis or follow-up helps guide clinical decision-making; for example, the diagnosis of uric acid stones may allow drug dissolution therapy over surgical intervention. These have led to the development of new imaging techniques, such as dual-energy CT, increasing our ability to delineate the stone composition (54).

#### 2.2.2 Renal impairment

Renal function injury is divided into chronic injury and acute injury. Clinically, most renal injuries belong to chronic injury. Most clinical symptoms are atypical, found during physical examination, and are more common in patients with long-term but not severe hyperuricemia. A small proportion of patients presents with acute kidney injury.

Acute kidney injury is frequently observed in patients with leukemia and lymphoma with rapid degradation of malignant cells during chemotherapy, and this syndrome has been clearly defined as acute tumor lysis syndrome. Less common causes include primary hyperuricemia due to hypoxanthine-guanine phosphoribosyltransferase deficiency (Lesch–Nyhan syndrome) or hyperuricemia due to decreased urate reabsorption in the proximal tubules; the latter can occur during exercise in patients with acute Fanconi-like syndrome or in patients with familial renal hypouricemia due to deficiency of URAT1 activity. Urinalysis in patients with acute uric acid nephropathy shows numerous uric acid crystals, but the absence of output due to obstructed nephrons may not be evident. Excess uric acid excretion can be demonstrated in many patients based on a uric acid/creatinine ratio (mg/mg) greater than 1 detected in random urine samples; in contrast, uric acid/creatinine ratios are below 0.60–0.75 in most other types of acute kidney injury (6). Other intracellular components are also released upon massive tissue breakdown (as occurs in tumor lysis syndrome) and are likely to cause hyperkalemia, hyperphosphatemia, and hypocalcemia (7, 8). Hyperphosphatemia may lead to acute kidney injury not associated with uric acid deposition (7).

The onset of chronic kidney injury is mostly insidious, and clinical symptoms do not appear until the middle age, at which time renal function has been damaged to varying degrees. Early proteinuria and microscopic hematuria gradually lead to increased nocturia and decreased urine specific gravity. Eventually uremia develops from azotemia. Overall, 40%–50% of patients have moderate hypertension, with blood pressure fluctuating between 150 and 180 mmHg and being generally controllable with antihypertensive drugs. Acute kidney injury is characterized by a rapid increase in uric acid and uric acid crystal accumulation in a short period of time, as well as urine polymorphous crystals, hematuria, leukocyturia, gradual oliguria, anuria accompanied by deterioration of the condition, and 24-h uric acid excretion decreased or normal.

Gout and hyperuricemia are involved in the development and progression of renal disease through uric acid and urate crystals. In turn, the occurrence and development of kidney disease will reduce the body’s ability to excrete uric acid; uric acid concentration in the body increases, aggravating hyperuricemia and forming a vicious cycle. This vicious cycle also involves hyperuricemia and renal hypertension, which have synergistic effects on renal function loss in addition to independent effects (55).

## 3 Risk factors for kidney injury in gout

### 3.1 Kidney stones

Current studies have shown that risk factors for kidney stones caused by gout and hyperuricemia include gout and hyperuricemia itself, higher serum uric acid levels and long duration of disease, obesity, as well as various complications and some environmental factors.

Hyperuricemia increases the risk of kidney stones. A recent large Korean cohort study showed a dose–response relationship between elevated serum uric acid levels and increased risk of kidney stones in apparently healthy men, with people with serum uric acid levels of 10.0 mg/dL having the highest risk of kidney stones (56). As the course of patients with gout progresses, the probability of stones increases. Liang et al. found that patients with renal stones had longer duration of hyperuricemia and gout, more gout flares, and higher levels of inflammatory markers in a multivariate regression analysis of 653 patients with gout (57).

An excessive body mass index (BMI) also promotes uric acid stone formation in patients with gout and hyperuricemia. Because a high BMI leads to increased levels of free fatty acids, enzymes associated with uric acid synthesis are altered, increasing uric acid synthesis and the risk of uric acid nephrolithiasis (58). Dyslipidemia was more common in patients with gout than in those with asymptomatic hyperuricemia. High-density lipoprotein-C is a protective predictor of serum uric acid levels in gout (59).

Hypertension in patients with gout also increases the incidence of kidney stones (60). In patients with hypertension and gout, the circulating blood pressure in the vascular wall continues to be above the normal values, which causes renal microangiopathy, reduced renal blood perfusion, tissue hypoxia, and impaired renal function. Decreased renal filtration of uric acid leads to formation of urate crystals. Patients with diabetes have been reported to have significantly higher urinary uric acid levels than non-diabetic people because insulin resistance and hyperinsulinemia result in impaired renal ability to excrete acid load and altered ammonium production, resulting in lower urinary pH, which predisposes patients with diabetes to uric acid stones (61, 62).

In addition, environmental factors in patients with gout can also affect the incidence of kidney stones, and studies have found that the prevalence of uric acid stones is 9% in factory workers working in hot environments, whereas the prevalence of uric acid stones is only 0.9% in workers working in the same field of work at standard room temperature. This may be associated with low urine output in patients in hot environments (63).

### 3.2 Decreased renal function

Risk factors for decreased renal function due to gout and hyperuricemia mainly include various characteristics of the disease itself and drugs used during attacks and uric acid lowering.

The duration of gout is an independent risk factor for renal dysfunction in patients with gout. Patients with older age, longer duration of disease, comorbid hypertension, and urate crystal deposition are more likely to have renal function impairment. Liang et al. found that patients with renal dysfunction were older and had longer duration of hyperuricemia by comparing variable differences in 637 patients who underwent estimated glomerular filtration rate (eGFR) calculation (57). One study divided all patients into three groups according to 24-h urinary uric acid levels by recruiting 461 patients with chronic kidney disease at stages 3–4. The clinical and biochemical characteristics of the patients with chronic kidney disease were collected for logistic regression analysis. Correlation analysis between relative mRNA expression levels of human UAT (hUAT) and human URAT1 and serum uric acid levels was also performed. Logistic regression analysis showed that serum uric acid, CHO, uKim-1/Cr, and uNgal/Cr were independent and multiple risk factors for hyperuricemic nephropathy. Moreover, relative hUAT and human URAT1 mRNA expression levels were significantly correlated with serum uric acid levels in patients with chronic kidney disease (64). A higher BMI was significantly and independently associated with an increased risk of new-onset hyperuricemia in patients with hypertension. Hyperuricosuria and hyperuricemia also play a role in acute kidney injury following the administration of radiocontrast agents (65).

One study showed that the eGFR decreased more significantly in patients with tophi than in those without tophi (-4.8 ± 14.5 vs. -0.7 ± 11.9 ml/min/1.73 m2/year, respectively; P = 0.039). The presence of tophi was significantly associated with a rapid decline in the eGFR (β = - 0.136; P = 0.042). Therefore, early diagnosis and appropriate treatment are essential to prevent further deterioration of renal function in patients with tophi, but the study consisted of patients registered in a single medical center, the size of the study sample was relatively small, and its authenticity needs to be verified in a larger population (66).

In acute gouty arthritis attacks, non-steroidal anti-inflammatory drugs (NSAIDs) are effective in relieving arthritis symptoms. However, patients with hyperuricemia receiving NSAIDs in the setting of a low GFR and low serum albumin have a higher incidence of acute kidney injury (67). The mechanism of non-steroidal anti-inflammatory drug-induced acute kidney injury is mainly divided into direct and indirect nephrotoxicity. The former is characterized by acute interstitial nephritis, and the latter by prostaglandin (PG) inhibition, and direct nephrotoxicity often occurs after immune injury with a low incidence, whereas indirect inhibition of PG is more common.

Uric acid-lowering therapy remains the recommended first-line treatment for patients with gout, and allopurinol is the earliest and most commonly used drug, first marketed in 1966. The second most commonly used febuxostat has been approved in France since 2008. The mechanism of action of both drugs is based on the inhibition of xanthine oxidase (68). Although urate-lowering therapies have been considered renoprotective against hyperuricemia (69), some studies have shown that although beneficial in patients with gout, they do not prevent the loss of renal function (70). A case/non-case study performed in France based on the World Health Organization’s VigiBase database between 1 January 2008 and 31 December 2018 found that acute renal failure was reported 5.7 and 3.3 times more frequently with febuxostat and allopurinol, respectively, in patients with gout (71).

## 4 Early biomarkers of gout kidney injury

In the early stage of gout renal damage, serum Cr, blood urea nitrogen (BUN), and other laboratory indicators were within the normal range; when these indicators were abnormal, irreversible renal damage occurred, and these indicators were easily affected by other factors. Early diagnosis is, therefore, particularly important for the prognosis of gout renal injury. However, there are few studies on this, and most indicators lack specificity, but these indicators provide a positive direction for future studies on early biomarkers of gout and kidney injury. In this paper, we enumerate some biomarkers that have had a diagnostic role in early renal injury in patients with gout in recent years (**Table 1**).

**Table 1:** Early diagnostic biomarkers of gout renal injury.

Biomarkers	Study subjects	Origin	Key point	Limitations	Reference (DOI)
Metabolites (Betaine and Biotin)	Uox-Ko mice	Plasma	Betaine and biotin visually described the metabolic characteristics of hyperuricemic mice, and the pathological changes in the kidneys of Uox-Ko mice used in the experiment were highly consistent with those in human hyperuricemic patients.	Metabolite results were not verified in gout patients.	10.1016/j.bbadis.2022.166374. Epub 2022 Mar 9.
Serum cystatin C (Cys C)	Patients with gout	Serum	Cys C concentration in blood is determined by GFR and is less influenced by other factors	Low specificity.	10.1089/jir.2021.0034. Epub 2021 Aug 25.
Neutrophil gelatinase-associated lipocalin (NGAL)	Pediatric patients with hyperuricemia	Urine	It can reflect the early tubular injury index in patients with gout.	Whether tubular injury plays a role in renal injury in mild hyperuricemia is unclear.	10.1007/s00467-013-2491-y. Epub 2013 May 15.
Kidney Injury Molecule-1 (KIM-1)	Patients with gout	Urine	KIM-1 is a sensitive marker of early tubular cell injury	Limited sample size, single center only.	10.1155/2019/6025804. eCollection 2019.
Interleukin-18 (IL-18)	Hyperuricemic patients/SD rats	Serum	These results suggest that inflammatory factors are involved in the development of renal damage induced by hyperuricemia	The association between this marker and renal impairment in hyperuricemic patients is uncertain	10.1016/j.biocel.2018.01.001. Epub 2018 Jan 2.

### 4.1 Metabolites (betaine and biotin)

It is well known that gout is a metabolic disease; therefore, metabolomic analysis can provide the entire organism field on metabolic pathway dysregulation at the systems biology level and identify potential biomarkers from hyperuricemia to gout. A Chinese study found that patients with hyperuricemia and gout had different significantly abnormal pathways compared with control patients using pathway enrichment analysis for hyperuricemia, with arginine metabolism appearing to play a key role (72).

Plasma metabolic profiling in a mouse model of hyperuricemia was constructed by knocking out the urate oxidase gene. Betaine and biotin were found to be associated with renal function, the best discriminators of gout kidney injury with high-predictive power (area under the curve = 97.2), and identified as potential plasma metabolic biomarkers for predicting urate nephropathy (73). These results revealed a potential pathogenic mechanism of gouty nephropathy. Investigating these metabolic pathways in humans may provide novel targets for therapeutic intervention in gouty nephropathy and for developing novel therapeutic strategies.

### 4.2 Serum cystatin C

Serum cystatin C (Cys C) is freely filtered, reabsorbed, and metabolized by the glomeruli but not secreted by tubular cells and is not affected by diet, muscle mass, sex, or age. It is, therefore, more sensitive and specific than BUN and serum creatinine in reflecting the GFR (74, 75).

Patients with gout have hyperuricemia, which leads to endothelial cell damage and vascular smooth muscle cell proliferation. Cys C may be a marker of renal impairment and inflammation (76). Yanqun et al. grouped 140 patients with gout and renal impairment according to their GFR. The serum levels of urea nitrogen, uric acid, creatinine, and Cys C were assessed. The diagnostic efficacy of serum Cys C was assessed with non-parametric receiver operating characteristic analysis. The serum Cys C levels increased as the GFR decreased in gout and renal impairment. Serum urea nitrogen, uric acid, and creatinine levels were positively correlated with Cys C levels, showing that the serum Cys C level is a precise diagnostic marker for GFR and renal damage assessment and has a significant diagnostic value for renal damage in patients with gout (77). Honghutang et al. performed a retrospective analysis of the data of 47 patients with uncomplicated gout and 48 patients with gout complicated with early renal impairment and 50 healthy individuals as controls. Serum, univariate, and multivariate combined detection of early renal damage in patients with gout was performed. The results showed that Cys C and mALB were sensitive markers for the diagnosis of early renal damage. The combined diagnosis of CXCL16 and COX-2 can effectively improve the detection sensitivity of early renal damage in patients with gout. The reason is that combined detection can effectively compensate for the limitations of single index detection and effectively reduce the impact of other related factors on the index (78).

### 4.3 Neutrophil gelatinase-associated lipocalin

Neutrophil gelatinase-associated lipocalin (NGAL) is one of the most recently intensively studied renal biomarkers that can indicate early structural damage and patient renal prognosis (79). It is a protein with a molecular weight of approximately 25 kDa that can exist as a monomer or form homodimers with MMP-9 with a molecular weight of approximately 46 kDa or heterodimers with a molecular weight of approximately 135 kDa and belongs to the lipocalin family, a group of small secreted proteins involved in the transport of small lipophilic molecules (80). It is a promising biomarker for tubulointerstitial lesions.

JustynaTomczak et al. showed that urinary NGAL and KIM-1 levels were higher in children and adolescents with hyperuricemia than in normouricemic controls, and serum uric acid was positively correlated with urinary NGAL/Cr (81). Therefore, NGAL is sensitive to renal damage in gout and can be used as an early diagnostic biomarker for renal damage in patients with gout.

### 4.4 Kidney injury molecule-1

Kidney injury molecule-1 (KIM-1), a transmembrane glycoprotein, is a member of the immunoglobulin superfamily and differs from other members of its family in that it is expressed by both immunocompetent and epithelial cells. KIM-1–mediated cellular and humoral actions involve multiple physiological and pathophysiological processes (82).

Studies have demonstrated that urinary KIM-1, interleukin (IL)-1, IL-6, and tumor necrosis factor alpha are higher in patients with serum uric acid concentrations ≥ 6.0 mg/dL. High serum uric acid concentrations are associated with high urinary KIM-1 levels and are accompanied by increases in urinary pro-inflammatory cytokines (83). Zhang et al. divided 126 patients with gout into a gout with renal injury group (n = 54) and a gout without renal injury group (n = 72). Fifty healthy subjects were selected as a control group. Urinary KIM-1, NGAL, and IL-18 levels were observed. The results showed that urinary KIM-1, NGAL, and IL-18 levels in the gout with renal injury group were higher than those in the gout without renal injury group and the control group (P < 0.05), and urinary IL-18 in the gout without renal injury group was higher than that in the control group (P < 0.05). Receiver operating characteristic curve analysis showed that the area under the curve of KIM-1 was 0.927, sensitivity was 83.33%, and specificity was 88.89% (84). These results suggest that urinary KIM-1 in patients with gout and renal injury has a high diagnostic value for the early diagnosis of gout and renal injury.

### 4.5 Interleukin-18

The IL-1 family consists of 11 members containing the IL-1 consensus sequence A-X-D, where A is an aliphatic amino acid, X is an arbitrary amino acid, and D is an aspartic acid (85). It has been shown that IL-18 is of great significance in the early diagnosis of diabetic nephropathy (86) and lupus nephritis (87). Although IL-1β has a known critical role in gout, increasing evidence suggests that other IL-1 family members are also involved in the pathogenesis of hyperuricemia and gout flares (88).

In an animal study, Hu et al. observed increased levels of inflammatory cytokines IL-1β and IL-18 and aggravated kidney injury in a rat model of uric acid nephropathy. In further clinical trials, serum IL-18 and IL-1β levels were measured with enzyme-linked immunosorbent assay in 25 healthy controls and 30 patients with uric acid nephropathy, and IL-1β and IL-18 were found highly expressed in patients with uric acid nephropathy compared with healthy controls (89). These results suggest that inflammatory factors are involved in the occurrence and development of hyperuricemia-induced renal damage, providing a new theoretical basis for the study of prevention and treatment of hyperuricemic nephropathy.

### 4.6 Other early diagnostic methods

In addition to serum biomarkers, urinary microorganisms, i.e., the intestinal flora, are dysregulated in patients with gout, and genetic aspects can also be used as a means of early diagnosis of gout kidney injury.

#### 4.6.1 Microorganisms

Recent studies have found a dramatic decrease in microbial richness and diversity in the urine of patients with gout compared with healthy controls. Firmicutes and their derivatives, Actinobacteria and their derivatives, Prevotella species, and Corynebacteria were significantly enriched in in the urine of patients with gout. Receiver operating characteristic analysis showed that the top five altered microbial genera could be reliable markers to distinguish patients with gout from healthy individuals. These findings demonstrate that there are specific alterations in microbial diversity in patients with gout. Therefore, further investigation of the causal relationship between gout and the urine microbiome will provide new perspectives for the diagnosis, prevention, and treatment of gout (90).

The human gut microbiota is a complex community consisting of over 100 trillion microbial cells, which include more than 1,000 different species. High-throughput sequencing has greatly advanced our understanding of the gut microbiome. The gut microbiota is increasingly associated with the development of various metabolic diseases such as obesity, diabetes, dyslipidemia, kidney disease, and kidney stones (91). The richness and diversity of intestinal flora in patients with uric acid renal calculi were significantly lower than those in controls. Bacteroides and Fusobacterium are the dominant species that distinguish the gut microbiota of patients with uric acid renal calculi from that of the healthy population, and they are significantly positively correlated with serum uric acid levels. Fusobacterium has been shown to be mainly involved in the metabolism and degradation of certain short-chain fatty acids, amino acids, and sugars in UAS patients and plays an important role in inhibiting their synthetic pathways (92). Intestinal dysbacteriosis is also thought to play a key role in the pathogenesis of gout, and intestinal dysbacteriosis characterized by gout may become a non-invasive diagnostic tool for gout and a promising target for future preventive interventions (93).

#### 4.6.2 Genes

Genetic studies, particularly genome-wide association studies (GWASs), have identified approximately 40 loci reliably associated with serum uric acid concentrations in humans, which together explain approximately 7% of changes in serum uric acid concentrations, of which approximately 4% are explained by two major loci, SLC2A9 and ABCG2 (94). As mentioned earlier, the secretion-associated major transporter GLUT9 is encoded by the SLC2A9 gene. The non-synonymous Arg265His (rs 3733591) variant of SLC2A9 increases gout risk in some populations (95). The rs 16890979 polymorphism was associated with hyperuricemia in a GWAS of 6881 Koreans (96). In contrast, the rs 12510549, rs 16890979, and rs 1014290 polymorphisms prevent gout in Caucasians and/or Asians (97). The SLC22A12 gene is responsible for encoding URAT1, and a mate analysis suggested that the rs 475688 polymorphism in the SLC22A12 gene is associated with susceptibility to gout (98). This new molecular knowledge reveals new intervention targets for managing urate levels and preventing gout. Future studies should focus on large GWASs (including asymptomatic hyperuricemic individuals) and increasing the use of whole genome sequencing analysis to identify uncommon genetic variants with increased penetrance, providing more possibilities for clinical translation.

## 5 Conclusions and perspectives

In recent years, the incidence of gout has been increasing; 3.9% and 14.6% of the American population have gout and hyperuricemia, respectively (99). Hyperuricemia and gout are often imperceptible in the early stage of kidney injury. If gout kidney injury fails to be detected and treated in time, it is likely to lead to the formation of uric acid kidney stones, which are easily associated with chronic urinary tract infection and accelerate the progression of kidney injury. If hyperuricemia is accompanied by conditions such as hypertension and diabetes, the process of renal injury is faster. However, only one in five patients with acute gout was screened for chronic kidney disease within a month of presentation (100). Therefore, if early diagnosis and appropriate treatment can be provided, the degree of renal disease can be reduced or stopped.

Uric acid plays a critical role in the pathogenesis of both gout and gouty kidney injury. With the progress of GWAS and the study of various urate-related transporters, the mechanistic understanding of uric acid homeostasis has increased, and the genetic and acquired effects of serum uric acid concentration have also been more deeply understood. We should continue to investigate existing and possible pathways of uric acid homeostasis regulation so that we can gain a more comprehensive understanding of uric acid-related diseases and identify new therapeutic targets for drug discovery and development. For example, taurine has been shown to decrease UAT mRNA and protein expression in mice with hyperuricemia and may be a promising agent for the treatment of hyperuricemia (101). Other agents against URAT1, such as SHR4640 and RDEA3170, are still in clinical trials (102, 103), and sodium-glucose cotransporter 2 inhibitors are approved hypoglycemic agents that promote uric acid excretion by inhibiting uric acid reabsorption by the uric acid transporter, GLUT9 (104), which may also hold new promise for the treatment of hyperuricemia. Although a variety of biomarkers have been reported in the literature, none of the existing indicators are specific, and the discovery of new specific biomarkers is helpful for therapeutic breakthroughs and is important for the prevention and treatment of gouty nephropathy.

## Author contributions

SW wrote the manuscript. LZ, DH, LW, JL, QN, LM, XP collected the references. JG reviewed and revised the manuscript. All authors contributed to the article and approved the submitted version.

## Conflict of interest

The authors declare that the research was conducted in the absence of any commercial or financial relationships that could be construed as a potential conflict of interest.

## Publisher’s note

All claims expressed in this article are solely those of the authors and do not necessarily represent those of their affiliated organizations, or those of the publisher, the editors and the reviewers. Any product that may be evaluated in this article, or claim that may be made by its manufacturer, is not guaranteed or endorsed by the publisher.
